# Screening for post-traumatic stress disorder among adolescents following floods- a comparative study from private and public schools in Kerala, India

**DOI:** 10.1186/s12887-021-02933-4

**Published:** 2021-10-20

**Authors:** Geethu Mathew, Aby Dany Varghese, Ans Mary Sabu, Aleena Joseph

**Affiliations:** 1 ROHC (S)-NIOH, Indian Council of Medical Research (ICMR), Bangalore, Karnataka 562110 India; 2 Department of Paediatrics, K S Hegde Medical Academy, Nitte, Derlakatte, Mangalore 575018 India; 3 Believers Church Medical College, Kuttapuzha, Thiruvalla, Kerala 689103 India

**Keywords:** Kerala, Floods, Post-traumatic stress disorder, Adolescents

## Abstract

**Introduction:**

Disasters can have deep physical and psychological impact among survivors. An extraordinary southwest monsoon has unleashed floods and landslides in Kerala state in 2018. Adolescents are more vulnerable to psychological impairment after a disaster and trauma during initial stages of life can etch an indelible signature in the individual’s development and may lead to future disorders.

**Objectives:**

1. To screen for PTSD and associated factors among adolescents 8 months post floods in selected schools in flood-affected areas of Alleppey district of Kerala 2. To compare the proportion of adolescents screened positive for PTSD in public and private schools.

**Methodology:**

A 3-month, Cross-sectional study was done among 670 adolescents in private and public schools using stratified sampling in Alleppey district. The study tool included a structured questionnaire that collected information on sociodemographics, flood-related variables, Trauma screening questionnaire and academic performance.

**Results:**

The mean age of the participants was 16.03 ± 0.73 years with almost equal gender distribution. One-third of students reported flood-related damage to house/property, and a few lost their pets. Nearly 50% of the students reported that they still re-experience and get upsetting memories about flood events. The prevalence of probable PTSD noted to be 34.9%. We observed that 31% of students in public school screened positive for PTSD compared to 38.8% of private school students. (odds ratio = 1.409, CI 1.024–1.938). Male gender (Odds ratio = 1.503, CI 1.093–2.069), higher age (Odds ratio = 1.701, CI 1.120–2.585), damage during floods (Odds ratio = 2.566, CI 1.814–3.630), presence of morbidity (Odds ratio = 3.568, CI 1.888–6.743), camp stay (Odds ratio = 3.788, CI 2.364–6.067) and loss of pets (Odds ratio = 3.932, CI 2.019–7.657) were the factors significantly associated with PTSD. We noted a deterioration in academic performance in 45.9% of students who screened positive for PTSD.

**Conclusion and recommendations:**

High prevalence of stress disorder highlights the need for early identification and intervention for PTSD and including trained counsellors as a part of the disaster management team in future.

## Introduction

The American Psychiatric Association defines traumatic event as psychologically distressing, outside the range of usual human experience markedly distressing to almost everyone [1, 2]. Disasters, both natural and manmade cause community level traumatic reactions and can result in various physical and psychological effects on individual life in terms of personal and daily functioning [3, 4]. Previous systematic reviews have documented that post-traumatic stress disorder (PTSD) is the most commonly studied and reported psychiatric morbidity among survivors of natural disasters worldwide [1, 5, 6]. PTSD is a condition that arises as a delayed reaction to highly threatening or catastrophic situations characterised by persistent intrusive memories, persistent avoidance of stimuli associated with the trauma and persistent symptoms of increased arousal [7]. Eventhough PTSD can affect any age group, adolescents seem to be more vulnerable to psychological impairment after a disaster. Literature has consistently highlighted the impact of disasters among adolescents due to their particular stage of physical, psychological, social development and their dependency on adults. The prevalence of PTSD among children affected by disasters ranged between 14 and 56% among children living in different communities [3, 6, 8–12]. Following super cyclone in Orissa, 26.9% of adolescents had PTSD 1 year post-disaster and 28.6% had mild to moderate PTSD following Northridge Earthquake [13, 14]. .In a recent systematic review, the authors have stressed the correlation between trauma during the initial decades of life and psychological problems which continue for years. During a study in Nepal, the authors found that 32.7% of adolescents had PTSD 31 months after Earthquake [3, 5, 6, 15]. PTSD in adolescence is also associated with suicide, substance abuse, poor social support, academic problems, and poor physical health [16]. The effect on academic competence and underachievement in long run is a serious matter of concern. Students exposed to disasters can have problems with concentration, rational thinking, motivation, self-efficacy, and effort regulation, which can have harmful effects on their academic performance. Further exploration of this association in our setting needs due attention [9, 13, 17–19]. Similarly there could be other factors that are potentially associated with PTSD which are seldom explored. This includes socioecomic background and effect of flood related losses like loss/death of pets that are of high significance in the life of adolescents. It is important to highlight the fact that in Kerala, the type of school is an indirect indicator of Socioeconomic status and most of the students belonging to high socioeconomic status attend private school. Also the the kind of coaching they undergo, supervision, the environment they study, the type of social interaction they have is different in private and public schools which also could have a significant association with the psychiatric morbidity.

An extraordinary southwest monsoon has unleashed floods and landslides in Kerala state in August–October 2018, the magnitude of which has rarely seen in recent memory. Many districts in central Kerala, including Alleppey, were affected. To the best of our knowledge, there is no published literature on PTSD among adolescents from different socioeconomic strata and associated factors, including its impact on academic performance from Kerala. Also, there is a need for population-based studies across groups to assess the mental health impact on flood-affected populations to develop evidence-based early interventions tailored to the needs of that particular group. Evaluating for PTSD and associated factors would help in suitable and timely intervention preventing them from progressing to chronic psychiatric disorders. Hence this study was carried out with the following objectives.

## Objectives

1. To screen for Post-Traumatic Stress Disorder among adolescents 8 months post floods in selected schools in flood-affected areas of Alleppey district of Kerala.
2. To compare the proportion of adolescents screened positive for PTSD in public and private schools included in the study
3. To study the association between PTSD and selected factors (Socio-demographic variables, flood-related losses and academic performance) among adolescents

## Methodology

### Declaration of data

The datasets used and analysed during the current study available from the corresponding author on reasonable request. We ensured that all methods in this study were following relevant guidelines and regulations.

A Cross-sectional study was conducted from June – August 2019 in the Alleppey district of Kerala. The schools were converted to relief camps for floods in the study area, and it took nearly 6 months for the schools to restart their function. Being a school-based study, we had to wait till June 2019 to start data collection after finishing all formalities. Adolescents studying in high school and higher secondary classes of selected schools in flood-affected areas in the Alleppey district were included in the study using multistage stratified sampling (the strata being the type of school - Government and private). In a similar survey done in Uttarakhand, 32% of adolescents screened positive for PTSD [3]. Considering this prevalence and 5% precision, on calculation, our minimum sample size was to be 335. The total sample included in the study was 670 (335 each from 2 strata). The data was collected using a pretested semi-structured questionnaire which was divided into three parts.

#### Part 1: socio-demographic variables

- Socio-demographic variables- age, gender, occupation of parents, household size, type of family and place of residence.

#### Part 2: flood related questions, including the history of displacement/ stay in camps, morbidity during a flood event, damage to house/property in floods and loss of pets

Academic performance assessment – We obtained data on the students’ academic performance before and after floods from the class teachers, and we also studied the association of the performance with PTSD. Two methods assessed the academic performance.

1. Teachers’ subjective assessment regarding the students’ performance before and after the floods.
2. Comparison of average marks scored in the exam conducted before the floods with the marks obtained in the exam after the floods (more than 10% difference in the scores or change in grades)

#### Part 3 - Trauma screening questionnaire

Trauma screening questionnaire (TSQ- Brewin, 2002) TSQ is a brief validated tool designed to screen for PTSD. Each item is derived from DSM IV criteria and describes either a re-experiencing symptom of PTSD (1 TO 5 QUS) or an arousal symptom of PTSD (6 to 10). The 10-item scale gauges stress based on recall of mood and feelings; each item scored on a scale of 0 or 1, giving a total score ranging from 0 to 10. A cutoff score of 6 is considered positive for PTSD, and the tool has a sensitivity of 94% and specificity of 76%. The recommendation is that screening be conducted only after a minimum period of 1 month post-trauma to allow for normal recovery processes to take place [2, 11].

The authors obtained the Institutional Ethics Review Board (IERB) approval of Believers Church Medical College, Thiruvalla, Kerala, India for conducting the study. A pilot study was undertaken amongst a few adolescents in a nearby school before commencing data collection, and we made suitable modifications to the questionnaire. We approached the Principals and the management of the Schools for permission to conduct the study. Informed consent was obtained from the principal/guardian for those below 18 years, along with the student’s informed consent. On obtaining written informed consent and assent, pretested questionnaires were administered to the adolescents who fulfilled the inclusion criteria and data was collected by trained medical students.

### Statistical analysis

The data collected was coded and entered in Microsoft Excel. Epi info software was used for the statistical analysis. Descriptive statistics comprising of the mean (SD), frequencies and percentages were calculated. Proportions were compared using the Chi-square test. The estimates of risk were calculated using Odds Ratio with a 95% confidence interval. We considered a statistically significant *p*-value to be less than 0.05.

## Results

A total of 670 students (335 from Public and 335 from private) were included in the study. The mean age of the participants was 16.03 ± 0.73 years with almost equal gender distribution. The majority of the participants belonged to nuclear families and was hailing from a rural area. The median household size was 4 (IQR- 4,5). Socio-demographic variables in Table 1.

**Table 1:** Distribution of study population based on socio-demographic profile

Variable	Frequency	Percentage
1. Gender		
Male	308	46
Female	362	54
2. Class		
Class 10	104	15.5
Class 11	430	64.2
Class 12	136	20.3
3. Fathers Occupation		
Professional	162	24.1
Non Professional	456	68.1
Retired/Not working	52	7.8
4. Mothers Occupation		
Professional	170	17.9
Non Professional	170	25.4
Retired/Not working	380	56.7
5. Type of Family		
Nuclear	476	71
Joint	174	36
Extended/Others	20	3
6. Place of Residence		
Urban	292	43.6
Rural	378	56.4
7. Monthly Income		
< 10,000	231	34.5
10,000 – 50,000	275	41
> 50,000	164	24.5

### Flood related losses

Among the 670 participants interviewed, 189 (28.2%) reported having flood-related damage to house/property and 41, (6.1%) of them lost their pets. Few of them, 44 (6.6%), suffered from various illnesses after floods. The common morbidity reported was skin infection. Among students, 86 (12.8%) had to stay in relief camps during the flood event. Very few (6 of them) reported the death of a family member.

## Section 2: screening for post traumatic stress disorder among adolescents

Out of the 670 students studied, 234 (34.9%) were screened positive for PTSD 8 months post floods as assessed by the Trauma Screening Questionnaire. Nearly 50% of the students reported that they still re-experience and get upsetting memories about flood events at least twice a week. Some of them, 279 (41.6%), said they get disturbing dreams about the flood event. One-third of the study population experienced bodily reactions (fast heartbeat, sweatiness, dizziness) when reminded of the event and had sleep disturbances (difficulty falling or staying asleep). Some students also reported having difficulty concentrating after the event.

We compared the proportion of students who screened positive for PTSD across public and private schools. It was observed that 104 (31%) of students in public school screened positive for PTSD compared to 130 (38.8%) of private school students. (odds ratio = 1.409, CI 1.024–1.938). PTSD and its association with socio-demographic and selected flood-related variables were noted. Nearly 40% of male students studied screened positive for PTSD. In contrast, only 30% of the female students had PTSD during screening (Odds ratio = 1.503, CI 1.093–2.069). Similarly, higher age found out to be associated with PTSD. Among students > 15 years, 37.3% had PTSD compared to 25.9% among ≤15 years. (Odds ratio = 1.701, CI 1.120–2.585). Among students who stayed in relief camps, a high proportion screened positive for PTSD (Odds ratio = 3.788, CI 2.364–6.067). Another factor that found to be statistically associated with PTSD was flood-related damage. Students who reported flood-related damages had more risk of developing PTSD than those who did not report any damage. (Odds ratio = 2.566, CI 1.814–3.630). Among participants who lost pets, 65.7% had screened positive for PTSD. (Odds ratio = 3.932, CI 2.019–7.657). The presence of morbidity during and after floods was associated with PTSD (Odds ratio = 3.568, CI 1.888–6.743). Nearly two-thirds of students who reported the death of a family member in floods screened positive. However, the numbers were too small to make meaningful associations. Factors like parents occupation, family income, type of family and place of residence were not significantly associated with PTSD among the study population. Details in Table 2.

**Table 2:** Distribution of population-based on the prevalence of PTSD across groups

SLNO	Variable	Frequency and Percentage		CHI Square Value	*p* Value
		PTSD	NO PTSD		
1.	Gender				
	1. Male	123(39.9%)	185(60.1%)	6.29	0.012
	2. Female	111(30.7%)	251(69.3%)		
2.	Age				
	1.</=15 years	36(25.9%)	103(74.1%)	6.287	0.012
	2. > 15 years	198(37.3%)	333(62.7%)		
3.	Type of School				
	1. Public	104(31%)	231(69%)		
	2. Private	130(38.8%)	205(61.2%)	4.43	0.035
4.	Occupation of Father				
	1. Professional	52(32.1%)	110(67.9%)		
	2. Non-professional	160(35.1%)	296(64.9%)	1.82	0.402
	3. Others	22(42.3%)	30(57.7%)		
5.	Occupation of Mother				
	1. Professional	45(37.5%)	75(62.5%)		
	2. Non-professional	48(28.2%)	122(71.8%)	4.49	0.106
	3. Others	141(37.1%)	239(62.9%)		
6.	Monthly Income				
	1. < 10,000	83(35.9%)	148(64.1%)		
	2. > 10,000	151(34.4%)	288(65.6%)	0.157	0.692
7.	Place of Residence				
	1. Urban	93(31.8%)	199(68.2%)		
	2. Rural	141(37.3%)	237(62.7%)	2.155	0.165
8.	Damage in Floods				
	1.Yes	96(50.8%)	93(49.2%)	29.16	< 0.0001
	2.No	138(28.7%)	343(71.3%)		
9.	Loss of Pets				
	1.Yes	27(65.9%)	14(34.1%)	18.38	< 0.0001
	2.No	207(32.9%)	422(67.1%0		
10	Morbidity				
	1.Yes	28(63.65)	16(36.4%)	17.05	< 0.0001
	2.No	206(32.95)	420(67.1%)		
11	Death of a Family Member				
	1. Yes	4(66.7%)	2(33.3%)		
	2.No	230(34.6%)	434(65.4%)	2.68	0.101
12	Stay in Camp				
	1.Yes	32(37.2%)	54(62.8%)	33.708	< 0.0001
	2. No	404(65.1%)	180(30.8%)		

Out of the 670 students studied, 278(41.5%) showed lower academic performance post flood. The majority, 444 (66.3%) them complained of difficulty in concentrating after the flood. Among those who screened positive for PTSD, 45.9% found to have lower academic performance post floods.

## Discussion

This study attempted to explore the psychological impact of floods and possible factors associated with this condition among adolescents in Kerala. To the best of our knowledge, this is the only one and first of its kind exploring different unaddressed factors associated with PTSD which needs special attention during disaster preparedness in future. Post-Traumatic Stress Disorder is one of the lesser considered and studied, especially among adolescents. The study showed a high proportion of PTSD (34.9%) among adolescents even after 8 months post-disaster which ties well with the published literature. A similar study done by Nisha et al. in North India showed a prevalence of 32% among adolescents. Around 1 year after a super-cyclone, 26.9% of adolescents exhibited post-traumatic symptoms in Orissa [3, 13]. Literature has shown that adolescents are sensitive to psychological shocks. They are less well prepared than adults socially and psychologically for dealing with trauma. For these reasons, adolescents are more likely to develop PTSD. Kerala is one of the states which was seldom affected by natural disasters compared to other states in India. And this was the worst flood that has ever occurred in the state after 1924. Considering this is the first encounter of adolescents with floods of this severity, the high psychological impact in population is perspicuous. Along with the significant psychological impact on adolescents this study cast light on various demographic, socioeconomic and flood related factors which is found to have an association with the outcome variable ie PTSD. Hence it is worth discussing the interesting results revealed in the study by theorectically analyse the findings and come up with possible justification for each and every association. To start with sociodemographic variables, it was observed to have gender differences in prevalence with male preponderance. While the types of trauma adolescents experience may be the same, the review results have revealed differences in rates of PTSD by gender. Some studies found that males are more likely to experience trauma than females, which is consistent with our study findings [16]. Another factor that was significantly associated with PTSD was the age of the students. Available evidence suggests that adolescents belonging to higher age groups were more affected by a disaster than lower age groups. It was reported in literature that cognitive immaturity may protect younger age groups from appreciating the implications of a disaster and this may be a conceivable explanation regarding this [14, 20]. We had compared the prevalence of PTSD across private and private school students, and we observed a significant difference in the proportion of students screened positive for PTSD. Logical and plausible interpretation could be that private school students who belonged to comparatively high socioeconomic status may not be used to hardships compared to other students. Facing a disaster of this magnitude and staying in relief camps would be a different experience for most of them. Despite an extensive literature search, we could not find a study comparing PTSD among private and public-school students. At this stage of understanding we speculate that abose said could be the reason for the differences in the impact and future studies could fruitfully explore this aspect. Another seldom studied association in India is of school achievements and their association with PTSD. Lower academic performance was found in 45% of students who screened positive for PTSD post floods. A study done by Lafta et al. in Baghdad showed that nearly one-fifth of the students showed a deterioration in students’ academic performance post-disaster [20]. Published literature have shown that students exposed to trauma present with passivity, inability to concentrate, decreased IQ, motivation, reading ability, personal connections and “spacing out” which is essential for reading acahievement and inturn can affect the academic performance. In addition to that it is posulated that students cannot concentrate on their school work when they do not feel safe, and as a result are anxious, fearful, and focused on suppressing the traumatic memories [18, 19, 21, 22]. These findings are in accordance with our study results. A significant association was found between PTSD and flood-related stay in relief camps, the morbidity associated with flood events and flood-related loss in this study. A study done by Harikrishnan et al. in 2018 showed similar results with high stress observed among adolescents who stayed in relief camps. PTSD among adolescents following Tsunami was studied in Tamil Nadu by Prasntham et al. The study found out that children who were exposed to loss of life or property showed severe stress compared to others [23]. Another important yet highly ignored factor is the association between the loss of pets and its psychological impact among children and adolescents, an aspect which most of the studies have not addressed. It is interesting to note that even the loss of pets can have a profound psychological impact on adolescents. Our study observed a novel consequential association between the loss of pets during a flood event and PTSD. It is commonly understood that children and pets “develop strong bonds” as they often grow up together and share formative experiences. Hence the death of a family pet can trigger a sense of grief that is profound and prolonged and can potentially lead to subsequent mental health issues [24]. Results of this study go beyond the published evidence which warrants further investigations to validate these conclusions.

Strengths and Limitations: This study is among the few school-based studies that have looked into the post-traumatic stress disorder and associated factors following floods among adolescents in India. Stratified sampling with a good sample size involving a different type of school improved the study population’s representation. Even though the study tried to illuminate many uncharted areas, because of the cross sectional nature, it was difficult to establish a causal association between different factors and outcomes. As it was a school based study, it was not feasible to involve parents of the students in the current study. There is inadequate information on pre-disaster psychiatric morbidity, which is known to influence the post-disaster psychiatric morbidity. This study focused only on PTSD and presence of other disorders and co-morbidities is a likely possibility which cannot be ruled out. Ideally a comparative study with an unexposed control population will suggest the risk attributable to the disaster and secondary traumas associated with it.

## Recommendations

Along with various relief and post relief measures during natural disasters, this study stresses the need to identify and intervene early for post-disaster stress disorder in adolescent victims. A longitudinal followup study would yield better results to understand the long term impact especially the academic component. We want to highlight the need for trained counsellors in disaster management teams to perform an early screening and intervention for PTSD. Special focus on factors associated with stress will help in disaster preparedness, mitigation, response and rehabilitation of this vulnerable group.

## Conclusion

The results of this study underline the impact caused by floods on the mental health of adolescents. We noted that a considerable proportion of adolescents suffered from stress-related disorders even after 8 months of the disaster. A noteworthy observation was of higher stress among private school students. Increasing age, male gender, stay in flood relief camps, flood-related damages, morbidity and loss of pets were significantly associated with PTSD. Nearly 50% of the adolescents who screened positive showed deterioration in academic performance. These results indicate that PTSD is widely prevalent among the survivors reinforcing the need to develop effective, culturally sensitive interventions tailored to the population’s needs.

## Data Availability

Available on request from the corresponding author.
